# International Consensus Statement for recommended terminology describing hysteroscopic procedures

**DOI:** 10.52054/FVVO.13.4.037

**Published:** 2021-12-30

**Authors:** J Carugno, G Grimbizis, M Franchini, L ALonso, L Bradley, R Campo, U Catena, C de Angelis, A di Spiezio Sardo, M Farrugia, S Haimovich, K Isaacson, N Moawad, E Saridogan, T.J. Clark

**Affiliations:** Obstetrics, Gynecology and Reproductive Sciences Department, Minimally Invasive Gynecology Division, University of Miami, Miller School of Medicine. Miami, Florida, USA; 1 st Department of Obstetrics and Gynecology, Aristotle University of Thessaloniki, Tsimiski 51 Street, 54623, Thessaloniki, Greece; Demetra Infertility Center and Villa Cherubini Clinic, Firenze, Italy; Endoscopy Unit, Centro Gutenberg, Malaga, Spain; Cleveland Clinic. Cleveland Ohio, USA; Life Expert Centre, Leuven, Belgium; Department of Woman, Child, and Public Health, Fondazione Policlinico Universitario A Gemelli IRCCS, Rome, Italy; Department of Maternal and Child Health and Urological Sciences, “Sapienza” University of Rome, Rome, Italy; Department of Public Health, School of Medicine, University of Naples, “Federico II” Naples, Italy; Spencer Hospitals, Margate, United Kingdom; Hillel Yaffe Medical Center, Hadera, Rappaport Faculty of Medicine, Technion, Israel; Center for Minimally Invasive Gynecologic Surgery, Newton-Wellesley Hospital, Newton, MA; Division of Minimally Invasive Gynecologic Surgery, Department of Obstetrics and Gynecology University of Florida College of Medicine, Gainesville, Florida, USA; Reproductive Medicine Unit, Elizabeth Garrett Anderson Wing Institute for Women’s Health, University College Hospital, NWI 2BU London, UK; Birmingham Women’s and Children Hospital and University of Birmingham, Birmingham, B15 2TT, UK

## Introduction

Hysteroscopy is one of the commonest performed procedures in contemporary gynaecology and is considered the gold standard endoscopic procedure for the evaluation and treatment of women with intrauterine pathology (Gkrozou et al., 2015). Hysteroscopic procedures are conducted in a variety of health care facilities with or without the use of anaesthesia/analgesia or in a hospital operating room (theatre) with an anaesthetist in charge of pain management. The decision where and how to undertake hysteroscopic procedures depends upon a number of factors including the available infrastructure (staffing, equipment, facilities), preferences (both patient and clinician), the type of hysteroscopic procedure (i.e. feasibility, acceptability and effectiveness of diagnostic and operative procedures) and health economics (e.g. reimbursement, investment and cost-effectiveness). For diagnostic and simple operative procedures such as endometrial polypectomy, procedures performed outside of a formal operating room have been shown to be cost-effective, with a low complication rate, high rates of patient acceptability and patient’s satisfaction (Kremer et al., 2000; Saridogan et al., 2010; Moawad et al., 2014; Clark et al., 2015; Cooper et al., 2015). More complex and prolonged procedures, such as hysteroscopic myomectomy and lysis of dense adhesions, are generally conducted in a hospital operating room with an anaesthetist present.

The structure and delivery of health care services vary across national health care systems, with differing credentialing requirements, funding, mechanisms for reimbursement and laws and regulations. These differences have hampered a clear understanding of how contemporary hysteroscopic services are currently being delivered globally and how best to do this. Specifically, there is no consensus regarding the terminology used to describe the different hysteroscopic procedures, including the setting in which they are performed and the model of care (i.e. need for elective hospital admission and length of stay) of the patient undergoing hysteroscopic procedures. Terms such as “in-office”, “outpatient”, “ambulatory”, “day-case”, “in-patient”, “operating room” are used frequently interchangeably without standardised definition or common understanding.

There is, thus, a pressing need for the implementation of a common terminology to describe hysteroscopic procedures that can be used uniformly in clinical practice and research. The creation and adoption of a standard nomenclature will be helpful for clinicians and patients by allowing the quality and outcomes of clinical practice to be more robustly compared. Furthermore, research studies can be reliably compared, aiding the interpretation and generalisability of findings, as well as facilitating data syntheses (i.e. systematic reviews and meta-analyses).

Therefore, the American Association of Gynecologic Laparoscopists (AAGL), the European Society for Gynaecological Endoscopy (ESGE) and the Global Community of Hysteroscopy (GCH) formed an international working group of experts in hysteroscopy to develop a consensus statement of recommended terminology to use for describing different aspects of hysteroscopic procedures: (i) pain management, (ii) the setting where procedures are conducted, (iii) the model of care relating to the length of stay and need for admission, (iv) the type of procedure and (v) the approach to hysteroscopy.

## Methods

In June 2018, concerns about the lack of a standardised nomenclature to describe hysteroscopic procedures was identified by a group comprising of five expert hysteroscopists from Europe and the United States. The need to create a nomenclature that objectively and uniformly defined hysteroscopic procedures prompted this group to create a larger working group of leading international hysteroscopists to identify areas in which a standard nomenclature was lacking.

In December 2018, seventy gynaecologists with extensive experience in hysteroscopic procedures were selected to represent the international community. An email was distributed among the members of this group enquiring about identified areas of current hysteroscopic practice that, in their opinion, needed a common terminology. On January 31, 2019, a preliminary proposal regarding areas of hysteroscopic practice where standard nomenclature was needed, namely pain management, procedural setting, the model of care, and approaches to hysteroscopy, was drafted. This broad, preliminary consensus was presented and discussed at the Global Hysteroscopy Congress in Barcelona, Spain in June 2019, at the ESGE Annual Scientific Meeting in Thessaloniki, Greece in October 2019 and at the Annual Global Congress of the AAGL in Vancouver, Canada in November 2019.

Following this wide consultation, the AAGL, the ESGE and the GCH created an international working group of 15 experts in hysteroscopy with the objective of revising the preliminary draft and prepare a consensus statement for terminology to be used for hysteroscopic procedures. Each scientific organisation contributed with five members in the group including practicing clinicians and researchers who had demonstrated leadership and expertise hysteroscopic procedures.

A total of three online video meetings took place from April to June 2021 in which a final agreement for standardised nomenclature was obtained. The process for arriving at a consensus was as follows. Where there was consensus about terminology for a particular area of hysteroscopic practice, the statements were adopted and revised only for editorial reasons. Where consensus could not be achieved, the proposed statements were removed, and members of the working group had the opportunity to provide verbal and written comments, suggestions, and to propose changes. These statements were revised accordingly to encompass the views of the working group members. The revised statements including alternative statement options where applicable, were disseminated in advance of the next online meeting and subsequently discussed in that forum. If voting failed to reach a consensus, the same process was followed before and during the third and final online working group meeting. Where members could not attend the online meetings, their written opinions were sought in advance to allow formulation of consensus of the whole group.

## Results

The adoption and implementation of a common terminology to standardise reporting of hysteroscopic procedures was proposed to cover five domains; pain management, healthcare setting, model of care, type of hysteroscopic procedure and the hysteroscopic approach to the uterine cavity. A summary of the terminology is given in Table I, and more detailed descriptions are provided in the subsequent sections.

**Table I:** Overview of terminology for hysteroscopy.

Pain management	Level 1 Level 2 Level 3 Level 3(a) Level 3(b) Level 4 Level 5
Setting	Office Outpatient clinic Operating room
Model of care	Office Outpatient Ambulatory Extended Recovery Inpatient
Type of procedure	Diagnostic hysteroscopy Operative hysteroscopy
Approach of procedure	Vaginoscopy Speculum assisted

### 1. Pain management

Technological advances have led to the miniaturisation of hysteroscopes and ancillary instrumentation, which has facilitated the conduct of procedures without the need for anaesthesia or with the use of local genital tract anaesthesia alone. The feasibility of conducting procedures without the need for conventional general or regional anaesthesia is dependent upon several factors both clinical and non-clinical and these include the type of procedure, patient preferences, clinician expertise, the available instrumentation and infrastructure and how health services are reimbursed and regulated.

Thus, the management of pain is a key consideration when undertaking hysteroscopic procedures and needs to be clearly and consistently reported. A hierarchical description of pain management, consisting of five levels, is recommended. (Table II).

**Table II:** Levels of pain management used during hysteroscopic procedures*.

Level 1		No medication or the use of oral non-sedative medication
Level 2		Local anaesthetic to the genital tract
Level 3		Conscious sedation
	Level 3 (a)	Oral or inhalational medications with a sedative effect
	Level 3 (b)	Parenteral medications with a sedative effect
Level 4		Regional anesthesia
Level 5		General anaesthesia

Pain management should be defined according to the highest level of intervention used to control pain if combined therapies are used.

### 2. Setting

There is currently no common understanding on the best terminology to define the setting in which hysteroscopic procedures are carried out. Terms such as “in-office”, “outpatient”, “ambulatory”, “day-case”, “in-patient”, “operating room” are used without standardised definition and often incorporate both the place (facility) where procedures are conducted and whether patients are admitted and how long they stay for.

In order for a clearer understanding of the procedural situation, it is recommended that the setting is defined in alignment with the “International Association for Ambulatory (Day) Surgery (IAAS) Suggested International Terminology and Definitions” (International Association for Ambulatory Surgery, 2003) and according to the level of pain management that is feasible in the facility where the hysteroscopic procedure is performed (Table III). This categorisation recognises that the level of pain management is not related to the environment (hospital, surgical center, community clinic or office) where the hysteroscopic procedure is performed and is not dependent upon the need for admission or dictated by the planned length of stay.

**Table III:** Definitions of the setting for hysteroscopy.

Office *	The hysteroscopic procedure is performed in a medical practitioner’s professional premises where pain control up to level 3(a) can be administered.
Outpatient Clinic *	The hysteroscopic procedure is performed in a health care facility for the management of outpatients e.g. hospital, community clinic or a freestanding surgical centre where pain control up to level 3(a) can be administered.
Operating Room	The hysteroscopic procedure is performed in a fully equipped operating theatre where pain control up to level 5 can be administered

*In some countries, in compliance of local legislation, pain management up to level 3(b) can be administered in an office or outpatient room setting. In such exceptional circumstances, the setting can be described as office or outpatient clinic rather than operating room, but the reported clinical data should report that level 3(b) pain management was used including type of pain management administered and route of administration.

### 3. Model of care

To enable clarity over the definition of setting for hysteroscopy and ensure consistency with the IAAS definitions (International Association for Ambulatory Surgery, 2003), it is recommended that the need for admission, the length of stay and type(s) of facility should be used to define the “model of care” under which the hysteroscopic procedure is undertaken. (Table IV).

**Table IV:** Model of care for hysteroscopy.

Office *	The model of care will be considered as “office” when the patient arrives and leaves a medical practitioner’s professional premises, which provides an appropriately designed, equipped and serviced room(s), on the same calendar day.
Outpatient *	The model of care will be considered as “outpatient” when the patient arrives and leaves the facility (outpatient clinic / department of a hospital, community clinic or a freestanding surgical centre (public or private)) on the same calendar day.
Ambulatory	The model of care will be considered as “ambulatory” when the patient undergoing the hysteroscopic procedure is admitted to a facility (hospital or surgical centre) and discharged on the same calendar day.
Extended Recovery	The model of care will be considered as “extended recovery” when the patient is admitted to a facility (hospital or surgical centre) with discharge the following calendar day with a length of stay of less than 24 hours.
Inpatient	The model of care will be considered “inpatient” when the patient is admitted to a facility (hospital or surgical center) and discharged not sooner than the following calendar day, with a length of stay of at least 24 hours.

*In the United States the term office and outpatient are used interchangeably as regards the model of care.

### 4. Type of procedure

Hysteroscopic procedures can be diagnostic and / or therapeutic (operative). Diagnostic procedures aim to visualise the uterine cavity to detect or exclude endometrial and structural abnormalities (congenital and acquired) with or without tissue sampling (blind or directed biopsy). Operative procedures remove endometrial and myometrial uterine pathologies with the aim of providing a therapeutic benefit by alleviating gynaecological symptoms as well as allowing histological analysis of removed tissue. The suggested terminology recommended to distinguish the type of hysteroscopic procedure is shown in Table V.

**Table V:** Type of hysteroscopy.

Diagnostic hysteroscopy *	A hysteroscopic procedure to evaluate the uterine cavity / cervical canal with or without targeted biopsy (under hysteroscopic visualisation).
Operative Hysteroscopy **	A hysteroscopic procedure to treat uterine pathology, or symptoms arising from the uterus, under direct hysteroscopic visualisation using hysteroscopic instruments.

* The use of hysteroscopy is not intended for the evaluation and management of the patient with cervical cancer or its precursors.

** “Blind” intrauterine procedures, such as an endometrial ablation procedure without hysteroscopic visualisation, insertion of intrauterine hormonal devices etc. should not be considered an operative hysteroscopy according to the proposed classification. However, studies describing such uterine procedures should report pain management, setting and model of care as described in the preceding sections.

### 5. Approach of the hysteroscopic procedure

The traditional approach conducting hysteroscopy to access the uterine cavity consists of inserting a vaginal speculum to visualise the cervix, which is then grasped with a toothed forceps (tenaculum/vulsellum) to provide counter traction when dilatating the cervical canal and subsequently introducing the hysteroscope through the cervical canal and into the uterine cavity under direct visualisation. This technical approach was consolidated after the introduction of hysteroscopy to routine gynaecological practice in the 1990’s when procedures were generally conducted exclusively under general or regional anaesthesia. Advances in surgical technology, and in particular miniaturisation of endoscopes and ancillary instrumentation, has allowed hysteroscopy to be performed without the need for vaginal instrumentation because cervical dilatation is not routinely required. This progression allowed hysteroscopy to be undertaken without the requirement for an anaesthetist or a formal hospital operating room (theatre) and hysteroscopy conducted in more convenient and accessible office and outpatient clinic settings became established.

Once office/outpatient clinic hysteroscopy became established, some practitioners began using techniques which did not require conventional vaginal instrumentation with specula and forceps (Bettocchi and Selvaggi, 1997; Sharma et al., 2005; Cooper et al., 2010; Smith et al., 2019; De Silva et al., 2020). The hysteroscope was passed directly into the vagina, avoiding unnecessary pain induced by vaginal distension and manipulation/ traction of the cervix. Subsequent clinical trials and data syntheses have demonstrated the benefits of such approaches in terms of reducing pain and enhancing patient experience (Smith et al., 2019; De Silva et al., 2020).

It is recommended that the approach to hysteroscopy, namely how the cervix is visualized and accessed to enable entry into the cervical canal and uterine cavity, is defined according to whether vaginal instrumentation is used or not (Table VI).

**Table VI:** Approach to hysteroscopy.

Vaginoscopic	The hysteroscope is steered into the cervical canal without the use of a speculum and/or stabilising forceps to facilitate the visualisation of the cervix and entry into the cervical canal and uterine cavity.
Speculum assisted	The hysteroscope is steered into the cervical canal with the use of a speculum and/or stabilising forceps to facilitate the visualisation of the cervix and entry into the cervical canal and uterine cavity.

## Documentation and reporting

A proforma to report hysteroscopic procedures according to this nomenclature is presented in Table VII.

**Table VII:** Proforma for documenting and reporting hysteroscopy*.

Setting		Pain management	
Office	□	Level 1	□
		Level 2	□
Outpatient Clinic	□	Level 3 (a)	□
	□	Level 3 (b)	□
Operating Room	□	Level 4	□
		Level 5	□
Approach		Type	
Vaginoscopic	□	Diagnostic	□
Speculum Assisted	□	Operative	□
Model of care			
No admission		Admission	
Office procedure	□	Ambulatory procedure	□
Outpatient procedure	□	Extended recovery procedure	□
		Inpatient procedure	□

## Discussion

Hysteroscopy is considered the gold standard procedure for the diagnosis and management of women with intrauterine pathology and is one of the most common interventions in contemporary gynaecological practice (Gkrozou et al., 2015). Despite its ubiquity, there has been a lack of consensus when describing hysteroscopic procedures such that multiple terms are used across the international community without any clear definition as to what they mean. This lack of clarity has caused confusion and hindered reliable interpretation of clinical data and scientific communications pertaining to the practice of hysteroscopy. Moreover, robust comparisons of hysteroscopic procedures have been compromised, as have the ability to synthesise data in systematic quantitative reviews to help inform clinical practice. By producing standard nomenclature for 5 fundamentally important areas of hysteroscopic practice, we hope that practitioners will find this consensus statement relevant and easy to adopt when reporting hysteroscopic procedures in both daily clinical practice and research studies. To aid use of this nomenclature, a standard proforma has been provided to report hysteroscopic practice in a systematic way according to the pain management utilised, the procedural setting, the model of care adopted, the type of procedure and finally the approach to conducting the hysteroscopy (see Table VII). This reporting proforma is highly recommended to be used in publications to allow comparisons and future meta-analysis.

Contemporary hysteroscopy is performed in a variety of health care settings using different methods of pain control. Hysteroscopic procedures using smaller diameter endoscopes and improved operative technologies, are increasingly being performed without the use of general anaesthesia in variety of facilities. Avoidance of regional or general anaesthesia is associated with a decrease rate of complications, faster recovery times, high rates of acceptability, equivalent effectiveness and greater cost-effectiveness (Cooper et al., 2015; Diwakar et al., 2016). However, pain associated with such hysteroscopic procedures limits the feasibility of such procedures and can adversely affect patient experience (Zupi et al., 1994; del Valle et al., 2016; Paulo et al., 2016; Amer-Cuenca et al., 2020). Alternatively, the use of highly sedative medications, or regional/ general anaesthesia necessitates the presence of an anaesthetist and invariably a formal operating room. Thus, the working group agreed that in modern hysteroscopy it is important to clearly define and report the pain control measures adopted and the procedural setting. Consensus was reached about a hierarchy of pain control measures relevant to hysteroscopy and that the procedural setting should be defined according to the level of pain control used in conjunction with the type of facility where the hysteroscopy was undertaken. To allow greater clarity as regard to what was meant by procedural setting, the length of stay and plan for admission to a specific health care facility did not inform the definition of setting, but rather was kept distinct and reported within a separate “model of care” category the was separated

The working group felt that the type of hysteroscopic procedure should be dichotomised into diagnostic procedures and those where the hysteroscope was directly used to treat uterine conditions or remove intrauterine pathologies. Finally, the group believed that the approach to hysteroscopy, namely the technique to visualize and traverse the cervical canal to access the uterine cavity should be reported in a standard fashion. This was in recognition of the miniaturisation of endoscopes and ancillary equipment over time that has facilitated hysteroscopic procedures being performed without the need for any other vaginal instrumentation, namely vaginal specula or forceps applied to the intravaginal cervix (Bettocchi and Selvaggi, 1997; Sharma et a., 2005). These, “vaginoscopic” approaches are quicker and less painful (Smith et al., 2019; De Silva et al., 2020).

The strengths of this consensus statement include the initial wide, international consultation to identify key areas where common definitions or hysteroscopic practice were needed and the subsequent formation of a working group of practicing expert clinicians and researchers by the three leading organisations in the field. This group produced and finalised the standard nomenclature by consensus following several iterations of the proposed statements and classifications until overall consensus was reached. Weaknesses of our approach include that we did not conduct a systematic literature search to identify published literature pertaining to standardised reporting of hysteroscopy. However, our literature searches did not find such papers and our international panel of leading experts in hysteroscopy were unaware of any standard nomenclature. Despite the international make-up of the working group, not all geographic areas were represented. Furthermore, our consensus was not reached using a highly structured approach, such as adoption of a formal Delphi process (Jones and Hunter, 1995). These methodological weaknesses may limit the validity and utility of the recommended hysteroscopic terminology. However, a consensus method very close to the expert panel technique was used as clearly described in the material and methods session and we believe that the group had adequate international representation and enough clinical and research expertise, in hysteroscopy to be cognizant of the current variations in terminology and reporting within global clinical practice and the international, published medical literature.

We anticipate that wide, international adoption of this standard terminology will vastly enhance communication in the field of hysteroscopy. In turn this will facilitate better understanding of clinical practice, the conduct and feasibility of techniques, and the cost-effectiveness of hysteroscopic interventions. Clinical and research collaborations will be facilitated, and data syntheses supported to robustly inform clinicians and patients.

## Conclusion

Hysteroscopy is the gold standard technique for the evaluation and management of uterine disorders and is widely used in modern gynaecological practice. However, a clear definition and understanding of the terminology used to describe hysteroscopic procedures is lacking. The production of this international consensus statement for terminology to describe hysteroscopic procedures, covering pain management, setting, model of care, type of procedure and hysteroscopic approach, has the potential to enable more effective communication for both clinical and research purposes with the ultimate aim of improving patient care and clinical outcomes.

A summary of the standard terminology describing hysteroscopic procedures is provided in the appendix.

## Appendix - Recommended nomenclature for reporting hysteroscopy

(Summary of the five domains to be recorded when reporting clinical data relating to hysteroscopic procedures for quality assurance or research publications / presentations)

**Domain 1: Pain management**

**Pain management is defined according to a hierarchy of escalating pain management strategies (five levels) for hysteroscopic procedures.**

Level 1:		No medication or the use of oral non-sedative medication
Level 2:		Local anaesthetic to the genital tract
Level 3:		Conscious sedation
	Level 3 (a)	Oral or inhalational medications with a sedative effect
	Level 3 (b)	Parenteral medications with a sedative effect
Level 4:		Regional anaesthesia
Level 5:		General anaesthesia

* Pain management should be defined according to the highest level of intervention used to control pain if combined therapies are used.

**Domain 2: Setting**

**Setting is defined in alignment with the “International Association for Ambulatory (Day) Surgery (IAAS) Suggested International Terminology and Definitions” and according to the level of pain management that is feasible in the facility where the hysteroscopic procedure is performed (1).**

Office *	The hysteroscopic procedure is performed in a medical practitioner's professional premises where pain control up to level 3(a) can be administered.
Outpatient Clinic *	The hysteroscopic procedure is performed in a health care facility for the management of outpatients e.g. hospital, community clinic or a freestanding surgical centre where pain control up to level 3(a) can be administered.
Operating Room	The hysteroscopic procedure is performed in a fully equipped operating theatre where pain control up to level 5 can be administered

* In some countries, in compliance of local legislation, pain management up to level 3(b) can be administered in an office setting. In such exceptional circumstances, the setting can be described as office or outpatient clinic rather than operating room but the reported clinical data should report that level 3(b) pain management was used and who and how it was administered

**Domain 3: Model of care**

**Model of care is defined according to the need for admission, the length of stay and type(s) of facility (in accordance with the IAAS (1) in relation to the hysteroscopic procedure undertaken.**

Office *	The model of care will be considered as “office” when the patient arrives and leaves a medical practitioner's professional premises, which provides an appropriately designed, equipped and serviced room(s), on the same calendar day.
Outpatient *	The model of care will be considered as “outpatient” when the patient arrives and leaves the facility (outpatient clinic / department of a hospital, community clinic or a freestanding surgical centre (public or private)) on the same calendar day.
Ambulatory	The model of care will be considered as “ambulatory” when the patient undergoing the hysteroscopic procedure is admitted to a facility (hospital or surgical centre) and discharged on the same calendar day.
Extended Recovery	The model of care will be considered as “extended recovery” when the patient is admitted to a facility (hospital or surgical centre) with discharge the following calendar day with a length of stay of less than 24 hours.
Inpatient	The model of care will be considered “inpatient” when the patient is admitted to a facility (hospital or surgical centre) and discharged not sooner than the following calendar day, with a length of stay of at least 24 hours.

* In the United States the term office and outpatient are used interchangeably as regards the model of care

**Domain 4: Type of procedure**

**Type of hysteroscopic procedure is defined according to whether it is diagnostic or operative, where it has therapeutic value:**

Diagnostic hysteroscopy *	A hysteroscopic procedure to evaluate the uterine cavity / cervical canal with or without targeted biopsy (under hysteroscopic visualisation).
Operative Hysteroscopy **	A hysteroscopic procedure to treat uterine pathology, or symptoms arising from the uterus, under direct hysteroscopic visualisation using hysteroscopic instruments.

* The use of hysteroscopy is not intended for the evaluation and management of the patient with cervical cancer or its precursors.

** “Blind” intrauterine procedures, such as an endometrial ablation procedure, insertion of intrauterine hormonal devices etc. without hysteroscopic visualisation, should not be considered an operative hysteroscopy according to the proposed classification. However, studies describing such uterine procedures should report pain management, setting and model of care as described in the preceding sections.

**Domain 5: Approach of the hysteroscopic procedure**

**Approach to the hysteroscopy is defined according to whether vaginal instrumentation is used or not:**

Vaginoscopic	The hysteroscope is steered into the cervical canal without the use of a speculum and/or stabilising forceps to facilitate the visualisation of the cervix and entry into the cervical canal and uterine cavity.
Speculum assisted	The hysteroscope is steered into the cervical canal with the use of a speculum and/or stabilising forceps to facilitate the visualisation of the cervix and entry into the cervical canal and uterine cavity.
